# Antidepressant effects of total alkaloids of *Fibraurea recisa* on improving corticosterone-induced apoptosis of HT-22 cells and chronic unpredictable mild stress-induced depressive-like behaviour in mice

**DOI:** 10.1080/13880209.2022.2099429

**Published:** 2022-08-07

**Authors:** Zhongmei He, He Yu, Hong Wu, Lili Su, Kun Shi, Yan Zhao, Ying Zong, Weijia Chen, Rui Du

**Affiliations:** a College of Chinese Medicinal Materials, Jilin Agricultural University, Changchun, China; b Jilin Ginseng Academy, Changchun University of Chinese Medicine, Changchun, China; c Key Laboratory of Animal Production, Product Quality and Security, Ministry of Education, Jilin Agricultural University, Changchun, China

**Keywords:** CUMS model, network pharmacology, BDNF-MEK/ERK signalling pathway, NLRP3/caspase-1 signalling pathway

## Abstract

**Context:**

*Fibraurea recisa* Pierre. (Menispermaceae) (FR) is a traditional Chinese medicine known as “Huangteng.” The total alkaloids of FR (AFR) are the main active ingredients. However, the pharmacological effects of AFR in the treatment of depression have not been reported.

**Objectives:**

This study investigates the antidepressant effects of AFR by network pharmacology and verification experiments.

**Materials and methods:**

Compound-Target-Pathway (C-P-T) network of FR and depression was constructed through network pharmacology. *In vitro*, HT-22 cells were treated with corticosterone (CORT) solution (0.35 mg/mL), then AFR (0.05 mg/mL) solution and inhibitor AZD6244 (14 μM/mL) or BAY11-7082 (10 μM/mL) were added, respectively. The cell viability was detected by CCK-8. *In vivo*, C57BL/6 mice were divided into 5 groups, namely the normal group, the CUMS group, the AFR (400 mg/kg) group, and the 2 groups that were simultaneously administered the inhibitory group AZD6244 (8 mg/kg) and BAY11-7082 (5 mg/kg). Western blotting was used to assess the expression level of the proteins.

**Results:**

AFR could protect HT-22 cells from CORT-induced damage and increase the cell viability from 49.12 ± 3.4% to 87.26 ± 1.5%. Moreover, AFR significantly increased the levels of BDNF (1.3, 1.4-fold), p-ERK (1.4, 1.2-fold) and p-CERB (1.6, 1.3-fold), and decreased the levels of NLRP3 (11.3%, 31.6%), ASC (19.2%, 34.2%) and caspase-1 (18.0%, 27.6%) in HT-22 cells and the hippocampus, respectively.

**Discussion and conclusions:**

AFR can improve depressive-like behaviours and can develop drugs for depression treatment. Further studies are needed to validate its potential in clinical medicine.

## Introduction

Depressive disorder is a prevalent psychiatric illness characterised by loss of interest, reduced energy, and pathological guilt (Ménard et al. 2016; Malhi and Mann 2018). It is estimated that 5% of adults suffer from depression every year worldwide, with the highest incidence among young people. In high-income countries, about half of people with depression go undiagnosed or untreated. However, this proportion reaches 80%–90% in low- and middle-income countries (Herrman et al. 2022). So, far, the pathogenesis of depression includes the monoamine transmitter hypothesis, receptor hypothesis, hypothalamic-pituitary-adrenal axis, neurotrophic factor hypothesis, inflammatory factor hypothesis, and oxidative stress hypothesis. It is closely related to genetic factors and social factors. Depression is a multifactorial and multi genetic syndrome, in which symptoms can vary from patient to patient (Zhang and Cheng 2019). Currently, almost all antidepressants are monoaminergic agents, and they are far from ideal. First, poor medication adherence is one of the factors influencing the progression of treat-resistant depression. Approximately 50% of depressed patients will experience a second depressive episode, and 10%–20% of people with depression do not get relief and progress to treatment-resistant depression (Kong et al. 2021). Second, the single-target approach has been successfully applied to single targets, but it is not effective for multifactor and multigene diseases, such as depression (Zhang and Cheng 2019). Third, the current clinical medication has many side effects (Khawam et al. 2006; Nabavi et al. 2017; Wang et al. 2018; Ramic et al. 2020). Therefore, researchers are committed to developing drugs from traditional Chinese medicine (TCM) with multi targets and multi pathways and fewer side effects.

*Fibraurea recisa* Pierre. (Menispermaceae) (FR) is distributed in China and Southeast Asian countries, such as Vietnam and Laos. It is known as “Huangteng” in TCM. The rattan stem of FR has been commonly used to treat skin diseases, including various tinea infections, cutis itching, and skin ulcers, by the native minority people Yao, Zhuang, and Miao in the south of Yunnan Province in China. FR is recorded in the State Pharmacopoeia of the People’s Republic of China as a phytomedicine and has been used as a TCM to treat different diseases for thousands of years, including clearing-heart, relieving-toxin, and relieving-constipation, in the TCM theory. Alkaloids are the main active compounds in modern chemical analysis technology (Fu et al. 2007; Rao et al. 2009). The authors found that the total alkaloids of FR significantly affect central nervous system disease (Xing et al. 2018; Zhang et al. 2020) and have the potential to develop drugs for brain diseases. Fibrauretine, one of the alkaloid components in AFR, can alleviate the comorbidity of diabetic neuropathic pain and depression by inhibiting the expression of P2X7 receptors in the hippocampus (Shen et al. 2018). Moreover, berberine was found to promote BDNF expression and regulate chronic unpredictable mild stress (CUMS) mice depression behaviours and hippocampal neurons growth (Zhan et al. 2021). Thus, AFR has the potential for antidepressant effects.

This study investigates the antidepressant effects of AFR treatment. The potential mechanism of AFR treatment of depressive disorders was analysed by network pharmacology. The target protein of the AFR active ingredient and the target of depressive disorders were collected through databases. Protein-protein interaction (PPI) network, Gene Ontology (GO) enrichment analysis, Kyoto Encyclopaedia of Genes and Genomes (KEGG) pathways, and compound-target-pathway (C-T-P) network were constructed. This study explored the potential therapeutic targets of AFR in anti-depression and verified the antidepressant effects of AFR by establishing corticosterone (CORT)-induced HT-22 cells model *in vitro* and establishing an antidepressant-like effect in a mice model of depression induced by chronic unpredictable mild stress (CUMS) *in vivo.* These results provided a basis for further understanding and exploring the mechanism of AFR in the treatment of depressive disorders.

## Materials and methods

### Material

The AFR is extracted by the laboratory of the College of Chinese Medicinal Materials of Jilin Agricultural University. Dried and pulverised rattan stem of FR (Yunnan, China) was refluxed with 55% ethanol for 100 min, three times. The liquid-to-material ratio is 9.8:1. The filtrates were combined and concentrated. D101 macroporous adsorption resin was used for further purification. It is eluted to remove impurities by distilled water. It was eluted again with 40% ethanol and the liquid was collected. Finally, the filtrates were combined and concentrated. After cooling, the extracted solutions were freeze-dried into powder as AFR for use (Wang et al. 2018). Corticosterone (Aladdin); CCK-8, AZD6244 and BAY11-7082 (Good Laboratory Practice Bioscience, GLPBIO); C57BL/6 mice (male, 6–8 weeks old, 18–21 g) were purchased from Liaoning Changsheng Technology and Biology Institute [license no. SCXK (Liao) 2020-0001]. It complies with the Laboratory Animal Welfare and Ethics Committee of Jilin Agricultural University (No. 20201118001).

### Construction of network pharmacology

#### Alkaloids targets of FR

Alkaloid components in AFR have been shown to have excellent clinical effects (Zhang et al. 2020). Alkaloids with good absorption, distribution, metabolism, and excretion (ADME) were important indicators. Oral bioavailability (OB) is one of the most significant pharmacokinetic parameters in ADME processes (Yi et al. 2020). It indicates the ratio of the drug taken to the blood circulation. The substances with OB ≥30% were regarded to have high OB. The larger the OB value of active compounds, the more likely it is to become a clinical drug for the treatment of disease (Fang et al. 2020). The similarity between a compound and a drug that is already known can be expressed in terms of drug-likeness (DL). It represents that although the compounds are not a drug, there is the possibility of becoming a drug (Hu et al. 2018). The DL index is useful for the rapid screening of active compounds. The compounds with a DL index ≥0.18 were considered to have high druggability (Guo et al. 2019). Therefore, the two ADME indicators of oral bioavailability (OB) and drug-like properties (DL) were used as initial screening conditions to establish a database of the effective active ingredients of AFR by the Traditional Chinese Medicine Systems Pharmacology Database and Analysis Platform (TCMSP; http://lsp.nwu.edu.cn/tcmsp.php). The compounds of OB ≥30% and DL ≥0.18 as the most important evaluation indicators were selected for subsequent research. The names of proteins and their ID were searched on Uniprot (https://www.uniprot.org/).

#### Depressive targets and PPI analysis

“Depression” and “depressive” were used as keywords to search and obtain related targets through the database, including the OMIM (https://www.omim.org/), GeneCards (https://www.genecards.org/), DrugBank (https://www.drugbank.ca/), DisGeNET (https://www.disgenet.org/), and TTD (http://db.idrblab.net/ttd/). The disease protein was obtained after removing the redundant entries. The intersection targets obtained above were imported into the Search Tool for the Retrieval of Interacting Genes (STRING) database (https://string-db.org/). By selecting “*Homo sapiens*” as the species, PPIs with combined scores of >0.7 were screened for further research. PPI is the basis of cell function of the body and plays an important role in regulating physiological and pathological states of the body. Network connecting lines represent protein interactions, and node size, colour, connection length, and thickness represent the topology parameters of the node network (Huang et al. 2020). A confidence level of 0.7 was selected and the nodes were obtained which play a very important role in the network.

#### GO and KEGG pathway analyses

GO and KEGG pathway analyses were executed through the biological database DAVID 6.8 (https://david.ncifcrf.gov/). The target gene was imported, and the species was defined as “*PPI*” A threshold of *p* < 0.05 was set, and GraphPad Prism 8 was used for graphical visualisation analysis.

#### Construction of the C-T-P network

Based on the above results, Cytoscape 3.7.2 was used to construct the Compound-Target-Pathway (C-T-P) network. It applies to visualising biological pathways and intermolecular interaction networks. The multi targets and multi pathways of AFR for depression were revealed.

### Cell verification experiments

#### Cell culture

The mouse hippocampal neuronal HT-22 cells were cultured in a DMEM medium containing 10% FBS and 100 units/mL penicillin-streptomycin and maintained at 37 °C in a humidified atmosphere containing 5% CO_2_ and 95% air. When the cell density is about 80%, it can be passed to the next generation, and it was used for the subsequent experiments after two passages.

#### Cytotoxicities of AFR and CORT on HT-22

HT-22 cells were seeded onto a 96-well culture plate at a density of 5 × 10^3^ cells/well and grown at 37 °C for 24 h. The medium was replaced with 100 μL Dulbecco’s modified Eagle’s medium (DMEM) containing different concentrations of AFR solution (0.001, 0.0125, 0.025, 0.05, 0.1, 0.2, 0.4, 0.8, and 1 mg/mL) for 24 h. CCK-8 (10 μL) was added to each well after adding the drug. After incubation for 2 h at 37 °C in 5% CO_2_, the absorbance of each well was then measured at a wavelength of 450 nm with a microplate reader (Epoch2; American Burton Instruments Co., Ltd.). Cell viability (%) was calculated as follows: Cell viability (100%) = (A_drug_–A_blank_)/(A_control_–A_blank_) × 100%. Under the same method, 10 μL CORT solution of different concentrations (0.05, 0.1, 0.2, 0.3, 0.35, 0.4, 0.5, and 1 mg/mL) was added to the wells and cultured for 8 h. The CCK-8 method was used for detection.

#### Protective effects of AFR on CORT-induced HT-22 cell damage

Different concentrations of AFR (0.0125, 0.025, 0.05 mg/mL) were added to the cell wells and pre-protected for 24 h. After that, CORT (0.35 mg/mL) was added and cell viability was tested.

#### Verification test by adding inhibitors

HT-22 cells were seeded onto a 96-well culture plate at a density of 5 × 10^3^ cells/well and incubated at 37 °C for 24 h. The medium was replaced with 100 μL DMEM containing 5% FBS and CORT solution (0.35 mg/mL) as the CORT group, whereas the control group did not contain CORT. AFR (0.05 mg/mL) and CORT solutions were simultaneously added to the wells as the AFR group. AFR (0.05 mg/mL) solution and inhibitor AZD6244 (14 μM/mL) or BAY11-7082 (10 μM/mL) as the pathway verification group (AFR + AZD or AFR + BAY group) and CORT solution were added after 24 h. In this study, AZD6244 inhibits MEK1/2 with high efficiency and high selectivity (Yeh et al. 2007). BAY11-7082 is an inhibitor of Nod-like receptor protein 3 (NLRP3) inflammasome, which can inhibit NLRP3 activation and block downstream signal pathways from being transduced (Qiu et al. 2017). Cell viability was tested using CCK-8 like the above experiment.

#### Immunoblotting analysis

Phosphate-buffered saline was used to wash HT-22 cells after administration. Cell lysate (100 μL; radioimmunoprecipitation assay/phenylmethylsulfonyl fluoride, 100:1) was added to the wells and lysed after 30 s. The samples were collected and then centrifuged at 12,000 rpm for 5 min at 4 °C. Total protein concentrations were determined with the BCA assay kit (Beyotime Biotechnology; cat. no. P0010). After the concentration of the sample was adjusted to be consistent, it was thoroughly mixed with the loading buffer and inactivated in boiling water for 3 min. The inactivated protein sample was loaded together with the marker. After separation by 10% sodium dodecyl sulfate-polyacrylamide gel electrophoresis, the gel was transferred to a polyvinylidene fluoride (PVDF) membrane (80 V, 110 min on ice). After the transfer, the PVDF membrane was cut according to the size of the target protein, placed in a protein incubation box, blocked with 5% skimmed milk powder for 2 h, and then incubated overnight at 4 °C with the primary antibodies against the following: anti-BDNF rabbit pAb (Shenyang Wanlei Biotechnology Co., Ltd.; cat. no. WL0168; 1:300 dilution); rabbit polyclonal antibody against ERK1/2 (Proteintech Group, Inc.; cat. no. 16443-1-AP; 1:1000 dilution); rabbit polyclonal against phospho-ERK1 + ERK2 (Abways Technology, Inc.; cat. no. CY5277; 1:500 dilution); rabbit polyclonal against NLRPS (Abways Technology, Inc.; cat. no. CY5651; 1:500 dilution); anti-ASC rabbit pAb (Shenyang Wanlei Biotechnology Co., Ltd.; cat. no. WL02462; 1:500 dilution); rabbit polyclonal against caspase-1 (Abways Technology, Inc.; cat. no. AY403; 1:500 dilution); β-actin rabbit polyclonal antibody (Proteintech Group, Inc.; cat. no. 20536-1-AP; 1:2000 dilution). It was then washed three-times with Tris-buffered saline with Tween 20 (TBST) after the addition of the primary antibody. The secondary antibody (goat anti-rabbit IgG (H + L) HRP; Proteintech Group, Inc.; cat. no. AB0101) was added and then incubated at room temperature for 2 h. The membrane was washed with TBST, and enhanced chemiluminescence was used for luminescence imaging.

### Animal verification experiments

#### Drug administration and CUMS model

C57BL/6 mice (male, 6–8 weeks old, 18–21 g) were kept on a 12 h light/dark cycle and randomly divided into depressive-like behavioural mice induced with CUMS (*n* = 40) and healthy control group mice (*n* = 8). For the establishment of the CUMS model, mice were forced to take the following measures, including 24 h water deprivation, 48 h food deprivation, 12 h cage tilting (45 degrees), turning the day and night, 12 h noise, 24 h wet bedding, and cold swimming (4 °C) for 5 min and were maintained for 21 days. The CUMS procedure was performed as described previously with minor modifications (Li et al. 2017). Simultaneously, CUMS-induced mice (*n* = 40) were divided into five groups: CUMS group (treated with 0.9% saline; *n* = 8) as the model group, AFR + CUMS (*n* = 8) as the therapy group (AFR group), AFR + AZD6244 (8 mg/kg)+CUMS (*n* = 8) as the verification group (AFR + AZD group) (Yeh et al. 2007; Ullrich et al. 2018), and AFR + BAY11-7082 (5 mg/kg)+CUMS (*n* = 8) as the additional verification group (AFR + BAY group) (Jiang et al. 2017; Qiu et al. 2017). Mice were intragastrically administered one dose of AFR (400 mg/kg; the effective dose was 1/15 of the LD50, which is 6 g/kg; *n* = 8) (Xing et al. 2018) for 21 days, dissolved in a 0.25% sodium carboxymethylcellulose solution based on the preliminary experiments and published papers. The CUMS schedule was performed continuously.

#### Body weight (BW)

The BW of each group of animals was recorded with a weighing apparatus on day 0 (before the beginning of the experiments) and day 21.

#### Sucrose preference test (SPT)

Mice were adapted to a 1% (w/v) sucrose solution for 24 h. After adaptation, mice were deprived of food and water for 24 h. Mice were given two bottles containing 1% sucrose and plain water, respectively. The position of the two bottles was changed randomly to avoid place preference. Sucrose and water consumption (mL) were measured after 2 h. SPT (100%) = sucrose consumption/(sucrose consumption + water consumption) × 100%

#### Forced swimming test (FST)

Mice were placed individually into cylindrical tanks (30 cm height × 20 cm diameter) filled with water (24 ± 1 °C) for 6 min. The passive time (immobility and floating motionless) from the second minute to the sixth minute was recorded.

#### Tail suspension test (TST)

Mice were individually suspended by their tails in a cage with their heads 15 cm from the bottom of the cage on the 21st day. The passive time (immobility and hang still) from the 2^nd^ min to the 6^th^ min was recorded.

#### Sample collection

After the behavioural experiments, the serum was collected. Which, the serum was treated by centrifuging blood clots at 3500 rpm for 10 min and was frozen at −80 °C until analysis. Then, the mouse hippocampus was stripped and stored at −80 °C for immunoblotting analysis.

### Data analysis

Statistical significance was determined using one-way ANOVA followed by Tukey’s comparison tests. *p* < 0.05 was defined as significant. Data were expressed as mean ± standard deviation (SD). GraphPad Prism 8 was used for graphing, and Image J was used for image analysis.

## Results

### Prediction of FR targets

**Figure 1.**
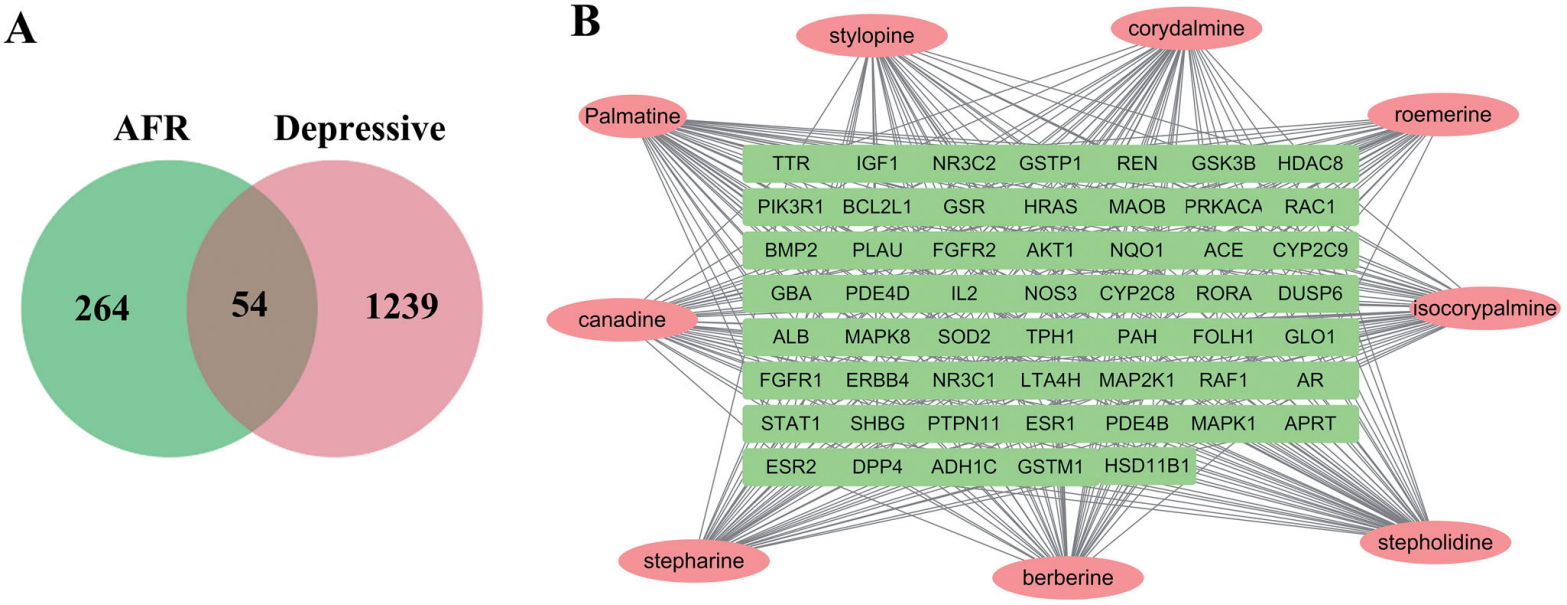
(A) Venn map of the intersection targets between the active components of FR and depression. (B) Network diagram of active ingredients-action targets.

Nine effective alkaloids were obtained after searching the TCMSP database. All selected OB and DL values of the compounds are shown in Table 1. The nine alkaloid ingredients are closely related to 264 targets after being processed by PharmMapper and Uniprot databases. Then, 1239 targets related to depression were obtained through OMIM, GeneCards, and other databases after removing redundancy. The component targets and disease targets were compared and matched, and a total of 54 genes were identified with Venn diagrams and are shown in Figure 1A. The 54 genes were both related to depression and FR.

**Table 1.** The effective active ingredients in FR.

No.	Molecule ID	Molecule name	PubChem CID	OB (%)	DL
1	Mol000785	Palmatine	19009	64.6	0.65
2	Mol001454	Berberine	2353	36.86	0.78
3	Mol004196	Corydalmine	161665	52.5	0.59
4	Mol007218	Roemerine	119204	40.75	0.52
5	Mol012921	Stepharine	98455	31.55	0.33
6	Mol000627	Stepholidine	6917970	33.11	0.54
7	Mol000790	Isocorypalmine	440229	35.77	0.59
8	Mol002903	Canadine	34458	55.37	0.77
9	Mol004230	Stylopine	6770	51.15	0.85

The nine effective ingredients and 54 intersection targets were imported into Cytoscape 3.7.2, and the effective ingredient-action target network diagram was drawn, as shown in Figure 1B. The pink oval node represents the effective components of the 9 AFR, the green rectangular node represents 54 intersection targets, and the edges represent the correlation between the effective components and the target. This showed that one active ingredient could correspond to multi targets, and the same target can correspond to different active ingredients. It confirmed the characteristics of the multi-components and multi-targets of AFR.

### PPI network construction

**Figure 2.**
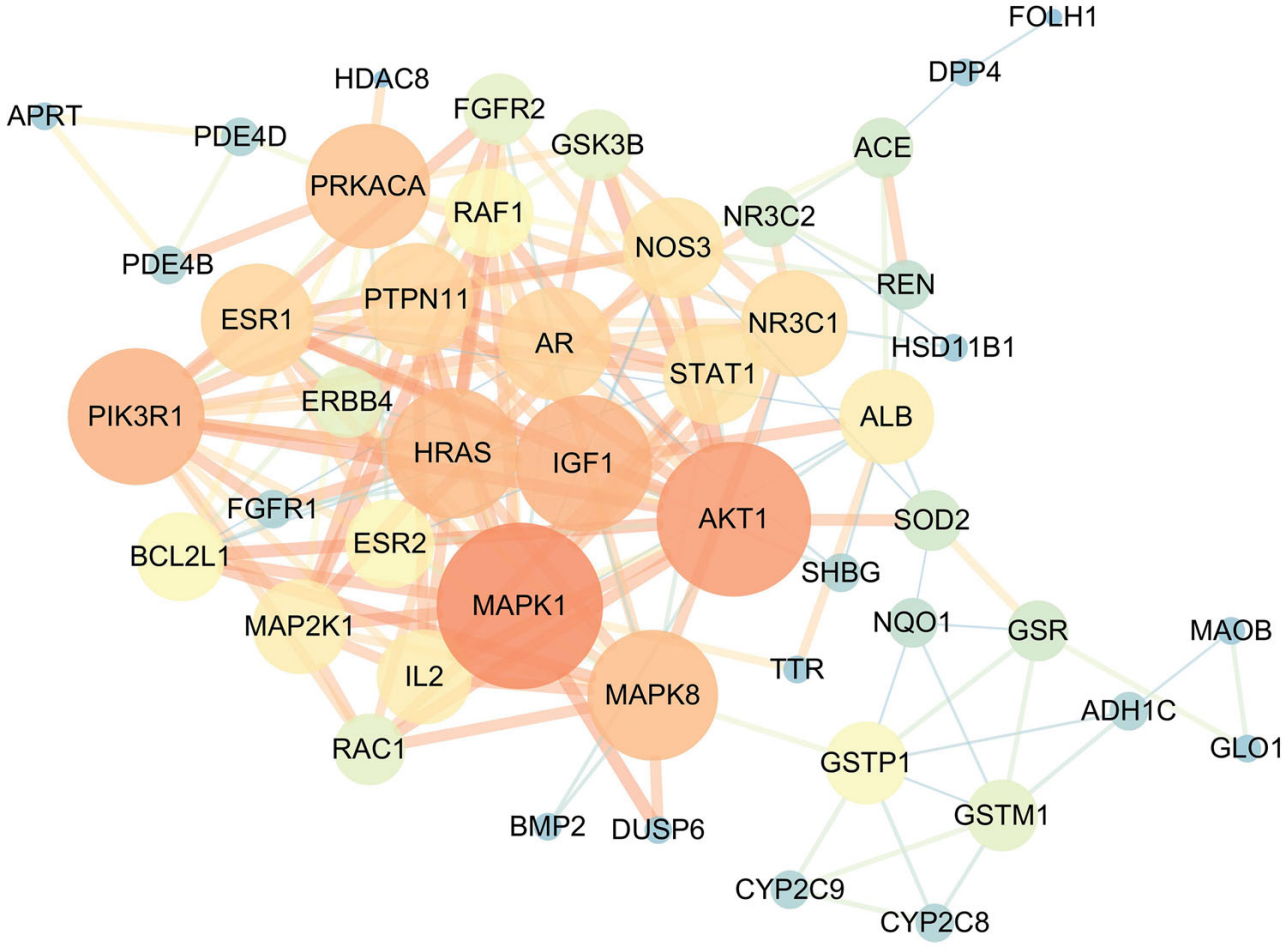
PPI network of the 54 overlapping genes.

The PPI network was constructed after 54 targets were imported into the STRING platform and visualised by Cytoscape 3.7.2. This node represents the protein, and the edge represents the correlation between the proteins in the network. As shown in Figure 2, a total of 48 nodes and 169 edges were finally obtained.

### GO and KEGG enrichment analyses

**Figure 3.**
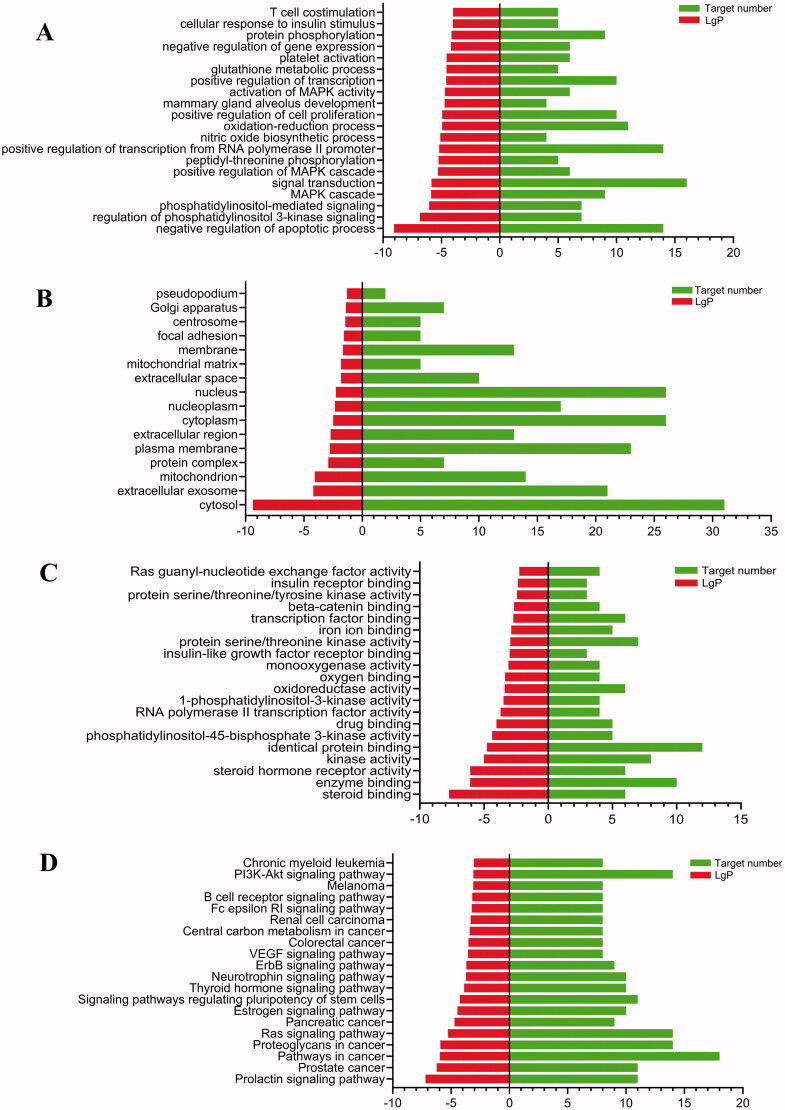
GO enrichment analysis: (A) BP, (B) CC, and (C) MF. (D) KEGG enrichment analysis.

The GO and KEGG enrichment analyses of the 54 targets were executed by DAVID 6.8. GO analysis included biological process (BP), cellular component (CC), and molecular function (MF) associated with BP and protein binding (Figure 3A–C). KEGG enrichment analysis (*p* < 0.05) obtained 34 signal pathways and are shown in Figure 3D. The results indicated that the effects of AFR on the treatment of depression were closely associated with the pathways, including phosphatidylinositol 3-kinase (PI3K)/Akt signalling pathway, Ras signalling pathway, and so on.

### C-T-P Network construction

**Figure 4.**
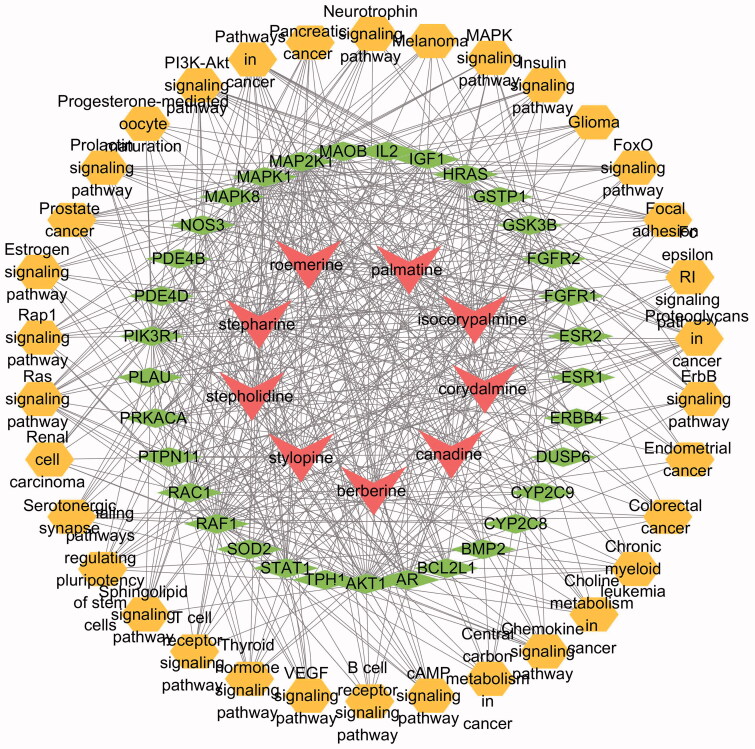
C-T-P network. The AFR compounds, target proteins and pathways are shown in red, green, and yellow, respectively. The edges show the connections of active ingredient-protein, protein-pathway and target-target.

The C-T-P network for AFR treatment of depression was constructed using Cytoscape 3.7.2 (Figure 4). The network comprised 76 nodes (9 compounds, 33 targets, and 34 pathways) and 541 edges. The green ellipse represents the compounds, the blue diamond denotes the potential targets, and the yellow circle represents the pathways. The results showed that these predicted targets were intensively associated with the following pathways: neurotrophin signalling pathway, mitogen-activated protein kinase (MAPK) signalling pathway, PI3K/Akt signalling pathway, FoxO receptor signalling pathway, etc.

### Verification of cell levels

#### Evaluation of cell viability

**Figure 5.**
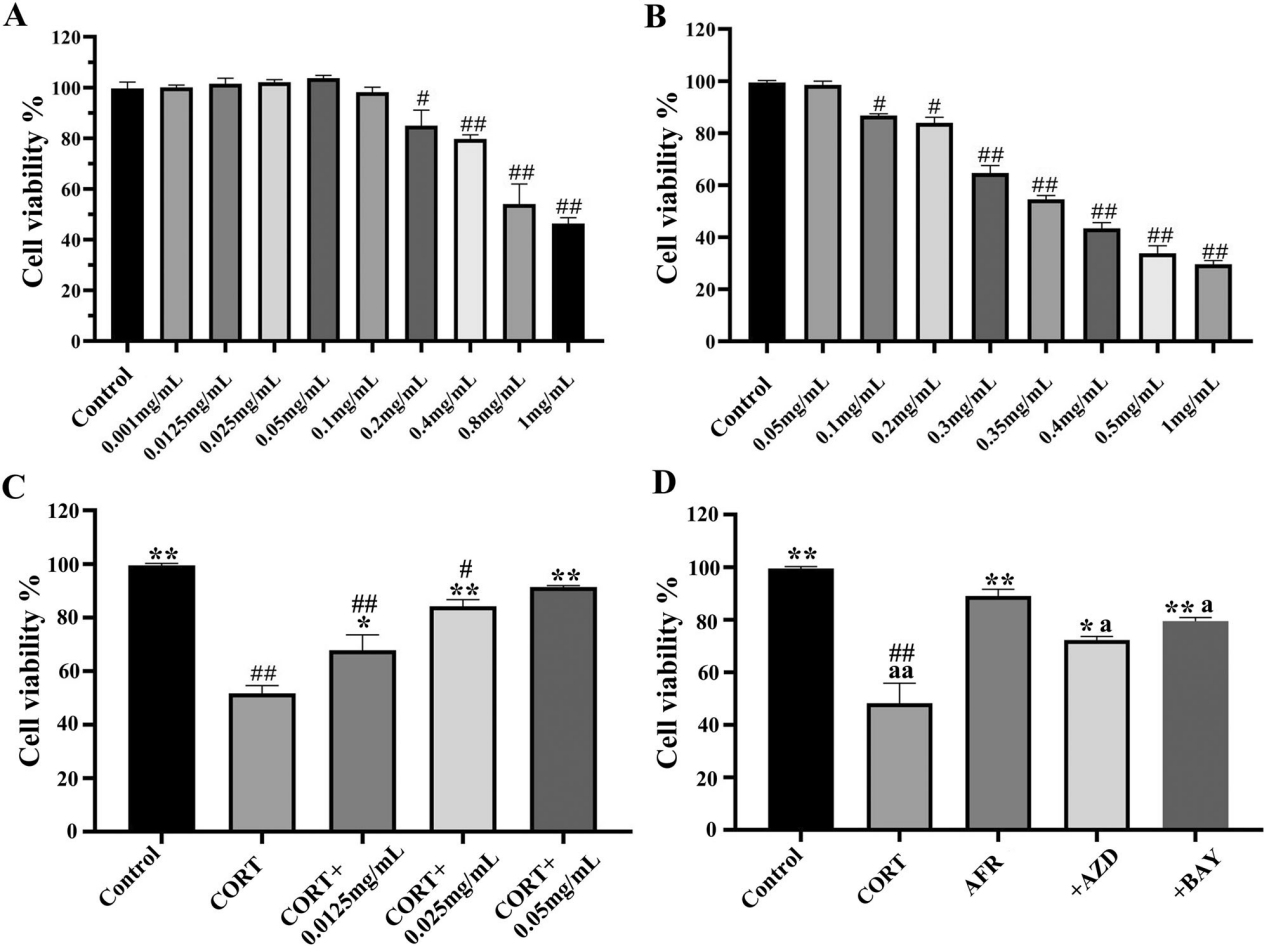
(A) Cell cytotoxicity of AFR at different concentrations in HT-22 cells using the CCK-8 assay. (B) Cell cytotoxicity of CORT at different concentrations in HT-22 cells using the CCK-8 assay. (C) Protective effects of AFR on CORT-induced HT-22 cells. (D) Effects of AZD6244 and BAY11-7082 inhibitors on cell viability after AFR treatment and CORT induction. *n* = 6, ^#^*p* < 0.05, ^##^*p* < 0.01, compared to the control group; *p < 0.05, **p < 0.01, compared to the CORT group; ^a^*p* < 0.05, ^aa^*p* < 0.01, compared to the AFR group.

The cell viability of AFR and CORT solutions was evaluated by the CCK-8 assay widely used to screen drugs. As shown in Figure 5A, the AFR concentration is greater than 0.05 mg/mL, and the cell viability gradually declined as the concentration increased in a dose-dependent manner, demonstrating that it was the appropriate concentration for evaluation experiments. The concentration of CORT-induced damage was 0.35 mg/mL (Figure 5B; the cell viability was 53.26 ± 0.12%), considered to be significantly different and suitable for subsequent drug evaluation. As shown in Figure 5C, AFR could protect HT-22 cells from CORT-induced damage.

Additionally, AZD6244 is a potent and selective inhibitor of both MEK1 and MEK2 (Yeh et al. 2007). It was added to the above solutions and used to verify whether the corresponding pathways could be recalled by AFR. BAY11-7082 is an inflammasome inhibitor, and studies have reported that BAY11-7082 could well inhibit NLRP3 inflammasome activation (Liu et al. 2014; Qiu et al. 2017). The results show that the AFR + AZD and AFR + BAY groups inhibited cell viability compared to the AFR group (Figure 5D). It suggested that the antidepressant effects of AFR may be through the MEK/ERK or NLRP3/caspase-1 pathway.

#### Effects of AFR on the expression of the brain-derived neurotrophic factor (BDNF)-MEK/ERK signalling pathway in HT-22 cells

**Figure 6.**
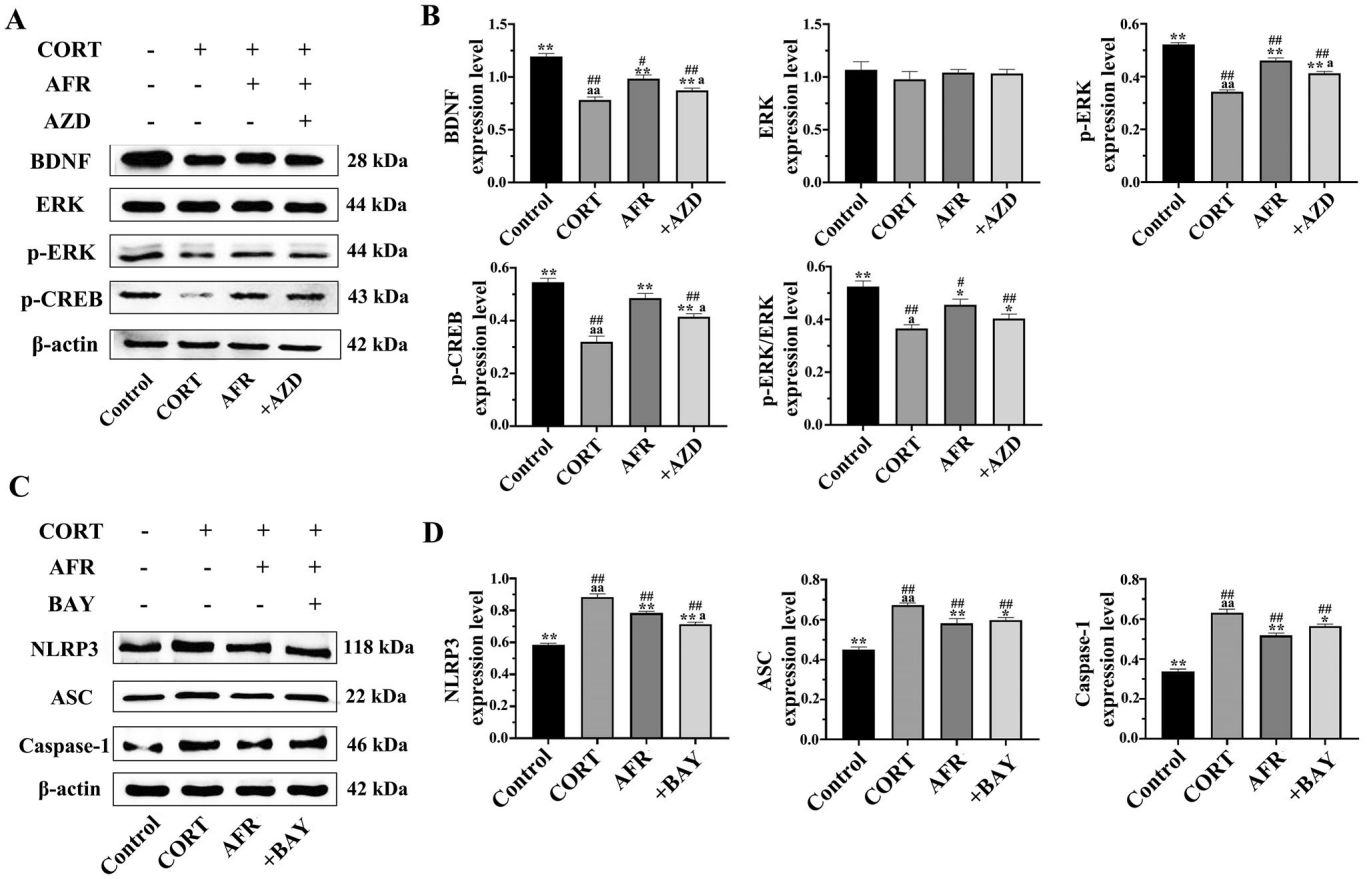
(A and B) BDNF, ERK, p-ERK and p-CERB expression in HT-22 cells was determined by Western blot analysis (mean ± SD; *n* = 3). Quantitative analysis was performed by densitometry. They were normalised to β-actin. (C and D) NLRP3, ASC, caspase-1 and β-actin expression were determined by Western blot analysis (mean ± SD; *n* = 3). Quantitative analysis was performed by densitometry. They were normalised to β-actin. ^#^*p* < 0.05, ^##^*p* < 0.01, compared to the control group; *p < 0.05, **p < 0.01, compared to the CUMS group; ^a^*p* < 0.05, ^aa^*p* < 0.01, compared to the AFR group.

The BDNF-MEK/ERK signalling pathway is one of the most important pathways in the nervous system, and it is closely related to neuroprotection and the treatment of depression (Pandya et al. 2013). Therefore, BDNF-MEK/ERK signalling in HT-22 cells was measured by Western blot analysis. As shown in Figure 6A and B, CORT significantly decreased the generation of BDNF, p-ERK, and p-CERB in HT-22 cells compared to the control group. AFR strongly promoted their production compared to the model group. BDNF and p-ERK expression were inhibited by AZD6244. It suggested that AFR significantly increased the BDNF-MEK/ERK signalling pathway in CORT-induced HT-22 cells, and AFR played a therapeutic role through the nervous signalling pathway.

#### Effects of AFR on the expression of the NLRP3/caspase-1 signalling pathway in HT-22 cells

Inflammation is an important factor in the aetiology and course of mood disorders and is related to inflammation of the central and peripheral immune systems. Especially, NLRP3 inflammasome may be a factor leading to depression (Kopschina Feltes et al. 2017; Adzic et al. 2018). As shown in Figure 6C and D, NLRP3, ASC, and caspase-1 expression were increased in the model group compared to the control group. Their expression was inhibited by AFR and the inhibitor BAY11-7082. It suggested that AFR significantly decreased inflammation in the signalling pathway in CORT-induced HT-22 cells. It may achieve therapeutic effects by inhibiting NLRP3.

### Verification of the animal levels

#### Behavioural influence of AFR in the CUMS model mice

**Figure 7.**
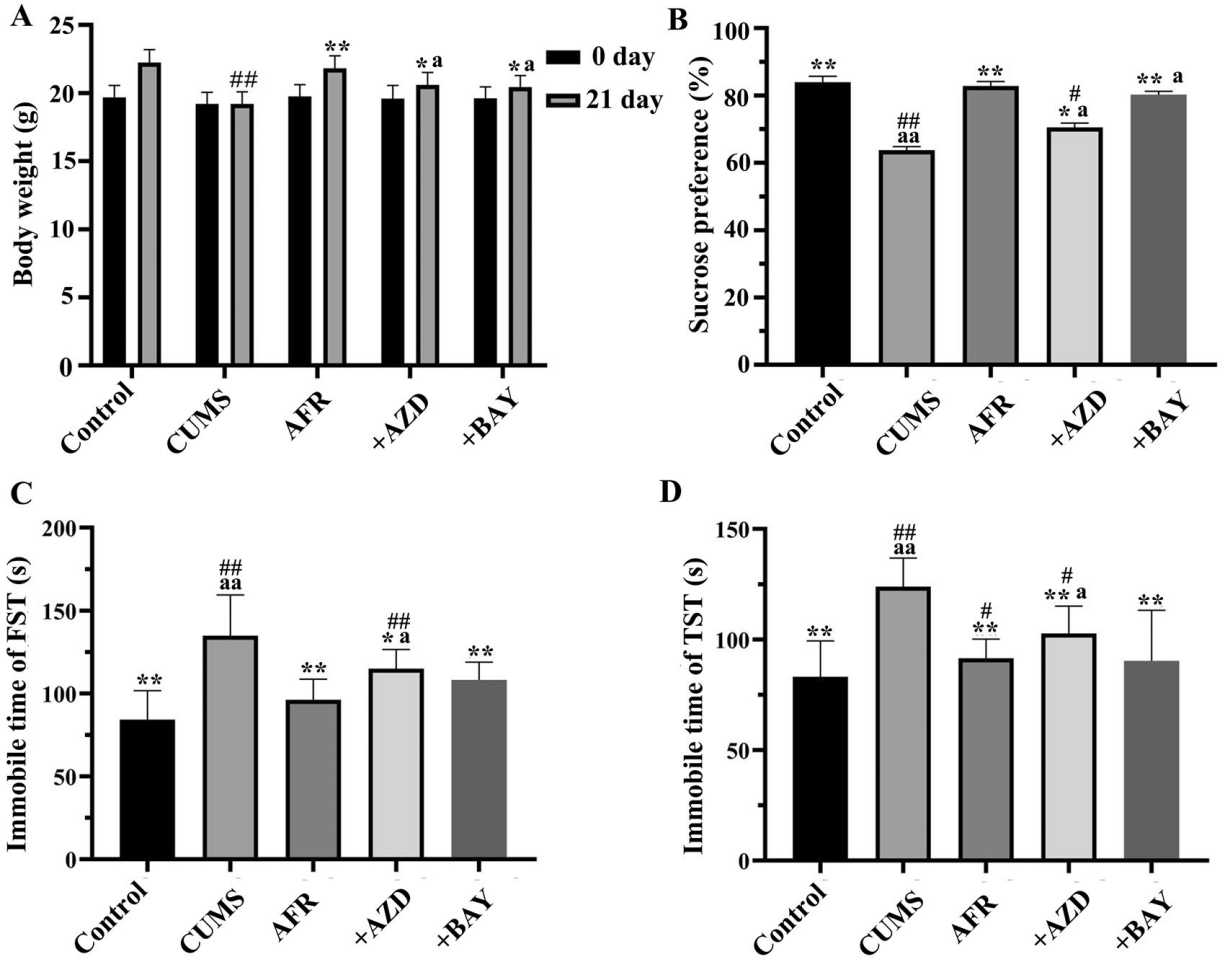
Behavioural tests were performed by (A) Body Weight (BW), (B) sucrose preference test (SPT), (C) forced swimming test (FST) and (D) tail suspension test (TST). *n* = 8, ^##^*p* < 0.01, ^#^*p* < 0.05, compared with the control group, **p < 0.01, *p < 0.05, compared to the CUMS group, and ^a^*p* < 0.05, compared to the AFR group.

Depressive-like behaviours were induced by CUMS and evaluated by BW, SPT, FST, and TST. As shown in Figure 7A, there were no differences in BW between groups before the experiment. After 21 days, the weights of the CUMS group significantly decreased compared to the control group (^##^*p* < 0.01), and the weights of the AFR groups significantly increased compared to the model group (**p < 0.01).

Next, the effects of AFR administration on the performance in SPF were examined. As shown in Figure 7B, the CUMS group was significantly decreased compared to the control group (^##^*p* < 0.01), and the AFR and AFR + AZD groups significantly increased compared to the CUMS group (**p < 0.01 or *p < 0.05). There was no significant difference between the AFR and AFR + BAY groups.

Similar to SPF, significant differences were obtained in FST and TST. As shown in Figures 7C and D, the immobility time of the model group was decreased by CUMS induction (^##^*p* < 0.01). The AFR and AFR + AZD groups significantly decreased compared to the model group (**p < 0.01 or *p < 0.05). There was no significant difference between the AFR and AFR + BAY groups.

#### Effects of AFR on the expression of the BDNF-MEK/ERK signalling pathway in the hippocampus

**Figure 8.**
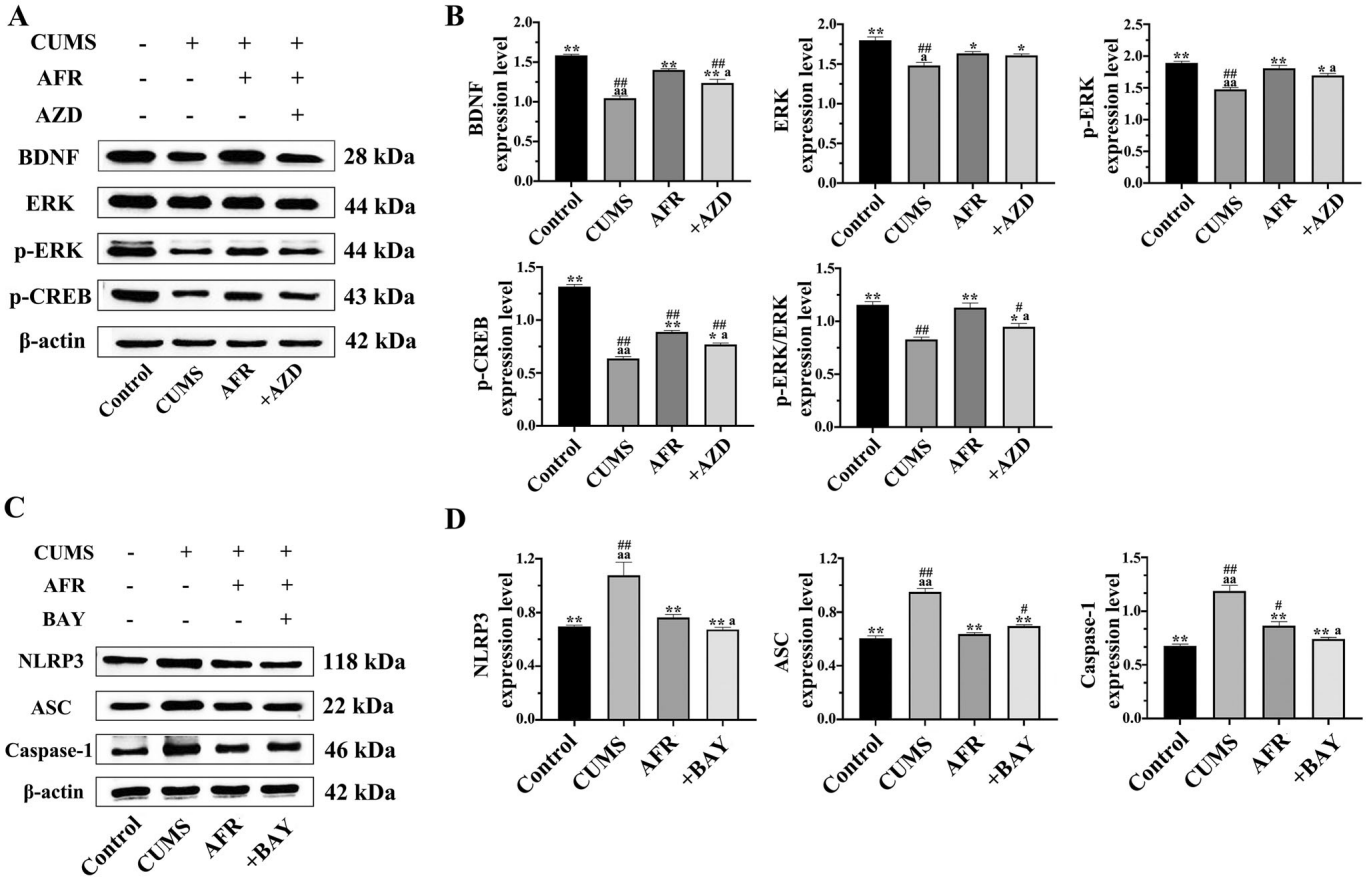
(A and B) BDNF, ERK, p-ERK and p-CERB expression in CUMS model was determined by Western blot analysis (mean ± SD; *n* = 3). Quantitative analysis was performed by densitometry. They were normalised to β-actin. (C and D) NLRP3, ASC, caspase-1 and β-actin expression were determined by Western blot analysis (mean ± SD; *n* = 3). Quantitative analysis was performed by densitometry. They were normalised to β-actin. ^#^*p* < 0.05, ^##^*p* < 0.01, compared to the control group; *p < 0.05, **p < 0.01, compared to the CUMS group; ^a^*p* < 0.05, ^aa^*p* < 0.01, compared to the AFR group.

Whether AFR could alter the BDNF-MEK/ERK signalling pathway in the hippocampus of the CUMS model was determined. As shown in Figure 8A and B, the model group significantly decreased BDNF, ERK, p-ERK, and p-CERB expression in the hippocampus compared to the control group (*p* < 0.05 or *p* < 0.01). The AFR and AFR + AZD groups strongly promoted their production (*p* < 0.05 or *p* < 0.01) compared to the model group. Compared to the AFR group, their expression in the AFR + AZD group decreased. It indicated that AFR significantly increased the BDNF-MEK/ERK signalling pathway in CORT-induced HT22 cells, and AFR played a therapeutic role through the nervous signalling pathway.

#### Effects of AFR on the expression of NLRP3/caspase-1 signalling pathway in the CUMS model

Similar to the cell model, significant differences were obtained among the groups for NLRP3, ASC and caspase-1 expression in the CUMS model. Compared to the control group, NLRP3, ASC and caspase-1 protein expression in the hippocampus of the model group was significantly higher than in the control group (*p* < 0.05 or *p* < 0.01) and are shown in Figure 8C and D. The AFR and AFT + BAY groups could significantly reduce NLRP3, ASC and caspase-1 protein expression than the model group (*p* < 0.05 or *p* < 0.01).

## Discussion

Depression is a disorder of mood, so mysteriously painful and elusive in the way it becomes known to the self. Evidence has accumulated over decades that depression is a leading cause of avoidable suffering in the world (Herrman et al. 2022).

Current antidepressants cannot completely cure depression status but can only alleviate the disease’s progress. For example, allopregnanolone, an allosteric modulator of the γ-aminobutyric acid type A receptor, is the first drug approved by the Food and Drug Administration to treat postpartum depression (Walton and Maguire 2019). Esketamine has shown significant pharmacological effects in phase III clinical trials by interfering with the cyclic AMP-protein kinase A-CREB signalling pathway and inhibiting the production of inflammatory factors, exerting an antidepressant effect (Lin et al. 2016). In addition, the first-line treatment of depression may include either a selective serotonin reuptake inhibitor or a tricyclic antidepressant. These drugs have the disadvantages of a single target (David and Gourion 2016; Molero et al. 2018; Rothmore 2020). Therefore, it is an urgent need to find antidepressants that can act on multi-targets from natural resources. TCM, with its unique curative effects in the treatment of depression, is attracting increasing attention. The concept of “multi-component, multi-target effects” has been widely applied to reveal the complex relationship between a herbal formula and the whole targets (Zhang et al. 2022). The rattan stem of FR is recorded in the State Pharmacopoeia of the People’s Republic of China in 2020. The main pharmacological effects of FR are anti-inflammatory and antibacterial (Ma et al. 2015). Alkaloids are one of the main active compounds in FR. However, there is no report on whether AFR could treat depression.

Network pharmacology was used to analyse the potential therapeutic effects of AFR on depression in this study. This field of systems network pharmacology analysis has developed rapidly in recent years. It reveals the complex analysis among multi components, multi targets, and multi pathways, especially for a complex compound of TCM (He et al. 2019; Zhang et al. 2019; Zhou et al. 2020). The molecular mechanism of TCM in treating diseases is analysed through network pharmacology, which provides new ideas and methods for the research of complex TCM systems.

This study explored the potential mechanisms of action of AFR against depressive disorders using a network pharmacology approach. Potential molecular targets of AFR and the interaction pathways were identified. There are nine potential antidepressant active ingredients, which act on 54 targets and play antidepressant effects through 34 related biological pathways. The results of GO and KEGG enrichment analyses indicated that depression involves a variety of biological processes in the body, including neurotrophic, vascular endothelial growth factor, PI3K/Akt, MAPK, and other signalling pathways.

Network pharmacology as a useful tool provides the complex biological information relationship between TCM and disease and helps further understand the effects of the drug (Zhou et al. 2020). Next, it is of great significance to verify the prediction results of biological network analysis through related experiments. The authors hope to verify a classic pathway and a less studied pathway. This study established CORT-induced HT-22 cells *in vitro* and CUMS-induced mice models *in vivo* to evaluate the antidepressant effects of AFR in the BDNF-MEK signalling pathway and NLRP3/caspase-1 inflammatory signalling pathway, respectively.

BDNF (neurotrophin) serves as a transducer and is closely related to the neurotrophic signal pathway of the central nervous system (Björkholm and Monteggia 2016). BDNF has profound effects on neuronal maturation, synapse formation, and synaptic plasticity (Yoshii and Constantine-Paton 2010). In addition, it can also act as a mediator of neuroplasticity changes between antidepressants and depression (Björkholm and Monteggia 2016). Studies have shown that serum BDNF levels are consistent with brain tissue BDNF levels, and BDNF levels can affect depressive-like behaviours in humans or animals (Schmidt and Duman 2010). Therefore, the increase or decrease of BDNF content can be used as an evaluation indicator of the antidepressant effects of drugs (Kuhlmann et al. 2017). The effects of most antidepressants are closely related to the up-regulation of BDNF levels and signal transduction pathways (Björkholm and Monteggia 2016). The BDNF-MEK/ERK signalling pathway is one of the most important pathways in the nervous system, closely related to neuroprotection and the treatment of depression (Pandya et al. 2013). BDNF binds to its receptor and activates Ras, allowing c-raf to bind to the plasma membrane. MEK is phosphorylated, which causes ERK1/2 phosphorylation at Ser217 and Ser221. The result is that the signal is transferred to the nucleus and CREB is activated (Liu et al. 2015). Activated CREB could also continuously promote BDNF expression so that the two form a cyclic response (Coyle and Duman 2003). Therefore, antidepressant drugs could increase BDNF expression and further regulate the expression of key factors, such as MEK, ERK and CREB, to exert antidepressant effects.

In the past decade, depression has been related to inflammation of the central and peripheral immune systems (Beurel et al. 2020). Especially, NLRP3 inflammasome is expressed in microglia and may play a crucial role in depression (Kopschina Feltes et al. 2017; Yue et al. 2017; Adzic et al. 2018; Feng et al. 2019). NLRP3 inflammasome can indirectly promote the production of interleukin-1β and other cytokines, thereby triggering a neurasthenic response. It is a potential new target for the development of antidepressant strategies. One study showed that the mechanism of antidepressants is the inhibition of the activation of the nuclear factor-κB pathway and NLRP3 inflammasome in the prefrontal cortex and hippocampus of CUMS rats (Wang et al. 2020). NLRP3 inflammasome is an inflammatory target of depression, and the pathogenesis of depression is closely related to neuroinflammation (Zhang et al. 2015; Su et al. 2017).

Therefore, based on the results of the network pharmacological analysis, CORT-induced HT-22 cells *in vitro* and CUMS-induced mice model *in vivo* were used to evaluate the antidepressant effects of AFR in the BDNF-MEK/ERK signalling pathway and NLRP3/caspase-1 inflammatory signalling pathway, respectively. This is of great value for AFR in the treatment of depression and drug development.

In *in vitro* experiments, AFR could protect CORT-induced HT-22 cells by cell viability. The inhibitors AZD6244 and BAY11-7082 were used to evaluate the ability of AFR to recall CORT-induced cell damage through the MEK/ERK or NLRP3/caspase-1 pathway, which can inhibit NLRP3 activation and block downstream. In *in vivo* experiments, the CUMS model is established by animals exposed to different kinds of mild stress every day, leading to depressive-like behaviours. The weights and sucrose preference of the AFR, AFR + AZD, and AFR + BAY groups significantly increased. The immobility time of FST and TST was decreased. It showed that AFR could improve the appetite and state of mice with depression and suppress depressive behaviours.

The expression levels of BDNF, ERK, and CREB in HT-22 cells and the hippocampus of mice were detected by Western blot analysis. The results showed that the protein levels of BDNF, p-ERK, and p-CREB in the model group were significantly lower than in the control group. The AFR and AFR + AZD groups can significantly up-regulate the expression of the above proteins. Compared to the AFR group, the expression of each protein in the AFR + AZD group had a downward trend, which was statistically significant. It showed that the antidepressant effects of AFR were related to improving the expression and function of key molecules in the BDNF-MEK/ERK-CREB signalling pathway. These antidepressant effects can be partially blocked by the MEK inhibitor AZD6244. It further illustrated that AFR exerts antidepressant effects through the BDNF-MEK signalling pathway. BDNF is synthesised in cell bodies of neurons and glia, which is related to synapse formation in the brain (Kowiański et al. 2018). The changes in BDNF fully prove that AFR could improve neuronal pathology in the depressed brain.

The results of the verification experiments of the inflammatory pathway showed that NLRP3, ASC, and caspase-1 expression was increased in the model group compared to the control group. Their expression was inhibited by AFR. It suggested that AFR significantly decreased inflammation in the signalling pathway *in vitro* and *in vivo*. It may achieve therapeutic effects by inhibiting NLRP3. To further explore whether the NLRP3/caspase-1 signalling pathway is involved in the process of the antidepressant effects of AFR, the effects of the NLRP3 inhibitor BAY11-7082 on CORT-induced HT-22 cell injury and CUMS-induced model mice were observed in this study. BAY11-7082 can inhibit the activation of NLRP3, block the downstream signal pathway from being transduced, and inhibit the appearance of its corresponding physiological and pathological effects (Liu et al. 2014; Qiu et al. 2017). Recent studies suggested that inflammation is closely related to depression. Inflammation activation is also considered an important factor in the occurrence and development of depression in recent years (Berk et al. 2013; Kohler et al. 2016).

The results demonstrated that AFR could relieve depressive-like behaviours observed in the CUMS-induced mice model. AFR regulated the relevant pathways of depression through multi-component and multi-target actions. It proved that AFR has therapeutic effects on depression by verifying the BDNF-MEK signalling pathway and NLRP3/caspase-1 inflammatory signalling pathway *in vitro* and *in vivo*. It suggested a new drug for antidepression to explore an effective and safe alternative treatment.

## Conclusions

Taken together, this study has revealed the therapeutic effects of AFR on depression. However, the pathway of AFR treatment of depression and the deeper mechanism of its target proteins are still unclear. Regardless, the results provided convincing evidence that AFR could be considered a potential antidepressant. This will provide a theoretical basis for the development of drugs for depression. AFR may be used as an alternative therapy for depression in the future.
